# Smartphone-based nystagmus diagnostics: development of an innovative app for the targeted detection of vertigo

**DOI:** 10.1007/s00405-022-07385-9

**Published:** 2022-04-22

**Authors:** Sara M. van Bonn, Sophie P. Behrendt, Bhushan L. Pawar, Sebastian P. Schraven, Robert Mlynski, T. Schuldt

**Affiliations:** 1 Department of Otorhinolaryngology, Head and Neck Surgery, “Otto Körner”, Rostock University Medical Center, Doberaner Strasse 137-139, 18057 Rostock, Germany; 2 Faculty of Computer Science and Electrical Engineering, Institute of Automation, University Rostock, Albert-Einstein-Strasse 22, 18059 Rostock, Germany

**Keywords:** Digitalization, Healthcare, Diagnostic app, Otorhinolaryngology, Dizziness, Emergency medicine

## Abstract

**Background:**

In medicine, the symptom of dizziness is one of the most common multidisciplinary causes of emergency medical presentation. Attending physicians are often faced with difficult decisions when evaluating patients with dizziness. A rapid differential diagnostic decision must be made during the initial examination. The goal of this study, was to develop a smartphone-based app that can diagnose and qualify nystagmus. The app should enable differentiation between acute emergencies such as strokes ("central vertigo") and vestibular disorders ("peripheral vertigo") using and recognizing or analyzing the accompanying symptom "nystagmus".

**Materials and methods:**

This prospective study was conducted at the Department of Otolaryngology, Head and Neck Surgery "Otto Körner", Rostock (Germany). The experimental study design consisted of two test runs and two control runs. In the two test runs, nystagmus was tracked and evaluated by caloric and optokinetic stimulation, respectively, through a custom-developed app. Sensitivity and correlation were calculated for the app's application performance and compared under different experimental conditions.

**Results:**

The patient sample included twenty healthy participants with a mean age of 25.6 years (± 2.2 SD) who participated in the study. The overall sensitivity of detection of nystagmus averaged 82.14% in the optokinetic stimulation test trials. There is no correlation regarding specific subject data and sensitivity.

**Conclusions:**

The results of our experimental validation study show that a smartphone-based nystagmus app is a useful tool for vertigo diagnosis. The results of our analyses show that it is possible to diagnose nystagmus and determine shape or direction with the app.

## Introduction

Due to increasing digitalization, not only social life has changed, but also the requirements for modern healthcare. Within the last few years, new technological innovations have increasingly reached medicine and have already drastically changed the healthcare system in some cases. The intelligent linking of enormous datasets of data through increasingly powerful IT systems holds the possibility of drawing a dynamic and holistic picture of the health of each individual. Individualized therapy through artificial intelligence-supported diagnostic options would be imaginable [1–3].

Due to the development and proliferation of accessible smartphones, they are an integral part of our everyday world [4, 5]. Increasingly, however, they are also a common and rapidly evolving tool in clinical practice. Mobile diagnostic devices can provide medical care at any location and around the clock. Telemedicine systems can be seen both as a resource for an underserved population and as a platform for physicians to consult with their patients remotely. Patients, especially in rural areas can be reached. Affordable diagnostic and or follow-up tools are available. Numerous applications or medical apps are already available for use on smartphones that also aim to help clinicians perform a variety of tasks at the point of care. Many custom software applications can be used in monitoring disease in particular. In particular, new apps have already proven to be very useful in the specialty of ophthalmology and diabetology [6, 7]. Despite the large number of available programs, the evidence of these is unclear. In particular, the user's interaction with the information obtained raises questions about the regulation, validation, and safety of the different app. However, given the ubiquity and usability of smartphones, health-related apps also provide an exceptional opportunity for data collection and epidemiological studies.

The smartphone and new apps for everyday clinical use can add to the existing wealth of clinical resources by serving as evidence-based decision support tools and can help optimize procedures and reduce errors [3]. However, the integrity of the data collected, transmitted, or calculated is highly dependent on the application and, therefore, must first be tested in scientific studies [8].

The aim of this study was to develop a novel app that reliably supports an initial assessment of the leading symptom of dizziness. In general, numerous studies show that the diagnostic and decision-making process in patients with vertigo is highly error-prone [9, 10].

Physicians, and especially emergency medical services as frequent primary care providers, are often faced with difficult decisions when evaluating patients with dizziness. Deciding on a diagnosis and possible treatment approach for a specific cause is challenging. Often, patients' symptom descriptions are equivocal. Examination findings among possible causes overlap. The app should enable a differentiation between acute emergencies such as strokes ("central dizziness") and vestibular diseases ("peripheral dizziness") using the accompanying symptom "nystagmus" and detecting respectively analyzing it. The clinically clear distinction can have relevant dimensions. In this context, the time that elapses until the decision is made is an important component.

## Methods

### Study design and patient selection

The prospective study was conducted at the Department of Oto-Rhino-Laryngology, Head and Neck Surgery “Otto Körner”, Rostock (Germany). The experimental study design was approved by the Ethics Committee of the Medical University of Rostock (file number A2018-0073). The patient selection included twenty healthy participants between the ages of 20 and 30 years. The participants were informed in detail about the study procedure by the investigator and signed an informed consent form to participate in the study. Exclusion criteria are known diseases of the vestibular organ or the central nervous system, visual disorders, tympanic membrane diseases, post-tympanoplasty, congenital nystagmus, physical or cognitive impairment, or tumors in the head region. Socio-demographic data were collected in a medical history interview and reference was made to the exclusion criteria. An ear microscopy for examination and cleaning of the auditory canal was performed. The participant was then positioned on the stretcher. During positioning, care had to be taken to tilt the head forward by 30°. If necessary, interfering factors such as make-up around the eyes were removed.

### App

The app was developed in cooperation with the Institute for Computer Science at the University of Rostock. For the hardware of this project, only an Android-based mobile device was used, which is equipped with a good resolution integrated camera. The reason behind choosing the Android OS is the free licenses of the developing tools. The app is based on an eye-tracking algorithm for the Android® operating system. The development of the Android application was performed on a computer with the Android Studio software installed. The used programming languages are Java and C++. The mobile application is built on the top of OpenCV framework which offers both Java and C++ APIs to communicate with the optimized core image processing algorithms. No costs were incurred.

**Fig. 1:**
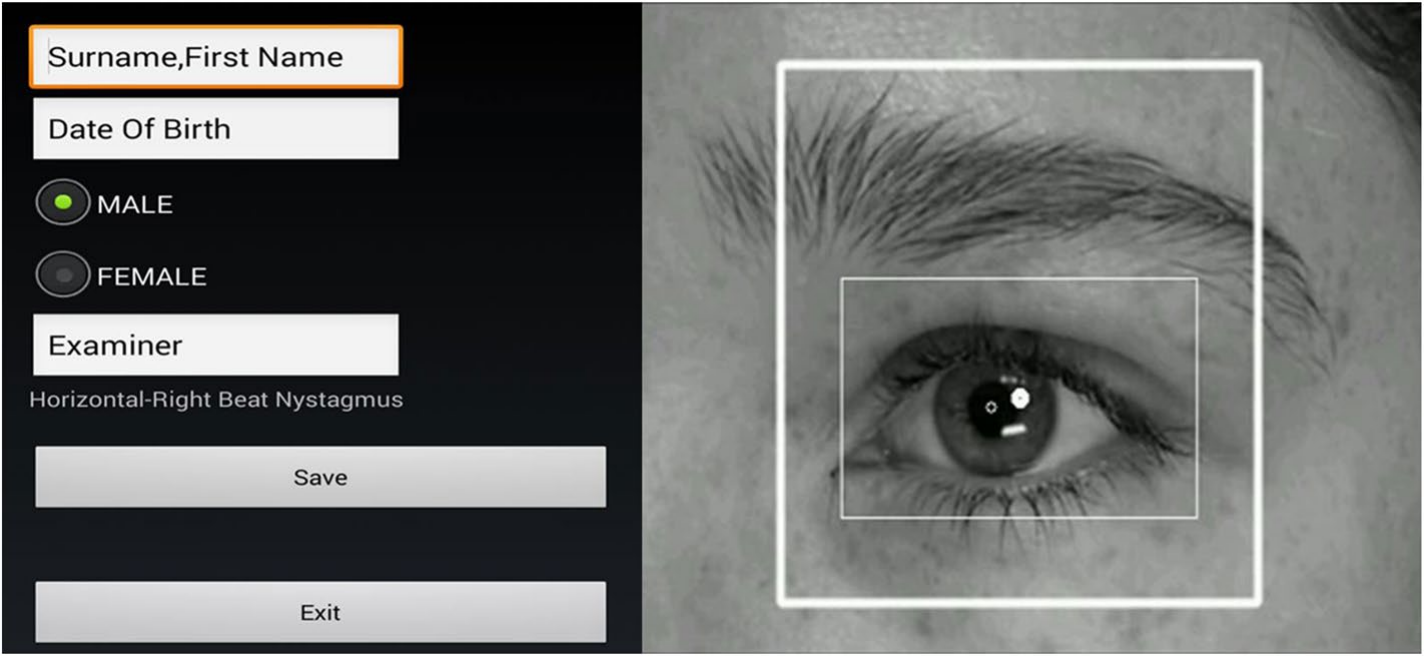
Data window for entering patient data and for the eye-tracking algorithm with marking of the region of interest

After entering the patient data (surname and first name, date of birth and gender of the patient as well as name of the examiner), the recording and evaluation of the pupil movement data began. The Android-based mobile device was always hand-held by the same examiner in front of the eyes. For data window delimitation, first the side to be examined is selected. Then, a hair cascade classifier is used to detect the region of an eye. To locate the center of an eye, an internal rectangle was positioned relative to the external rectangle for eye detection. The internal rectangle represents the region of the eyeball (Fig. 1). To detect the eye center, the algorithm of Timm and Barth was applied to the defined internal rectangle. The detected eye center is defined as a coordinate point, which is plotted on a line graph using the MP Android graph library. An encoding procedure was then developed to encode this line diagram into a binary format to predict the type of nystagmus. The eye-tracking method used here for low resolution images from web or cell phone cameras is that of Timm and Barth [11]. The algorithm uses the gradients of the image to track the eye. A function is derived that uses only dot products. The maximum of this function is the point where the gradient vectors intersect, which is the center of the eye.

**Fig. 2:**
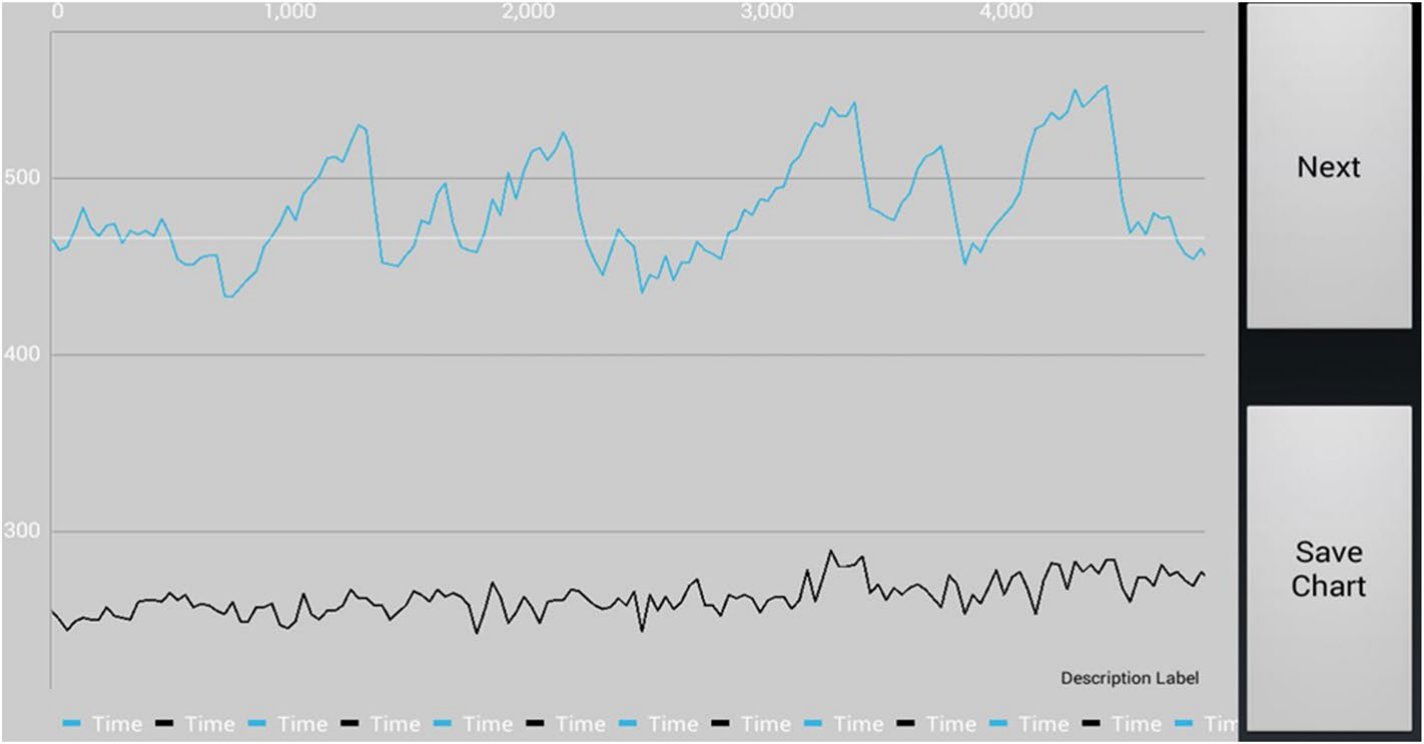
Result of app analysis for a horizontal right beating nystagmus (blue is the horizontal and black is the vertical component)

The measured motion data were mapped graphically as a ratio of distance to time. To reduce the amount of data and avoid artifacts, the algorithm is limited to the evaluation of sections with large pupil deflection. Thus, a distinction between horizontal and vertical nystagmus can be made. To evaluate up-beat and down-beat movements, the pupil deflections are coded using a binary code. Thus, it is detectable when a slow component (down-beat movements) in a nystagmus beat is always followed by a fast reset saccade (up-beat movements). The algorithm follows in the last step the command to find just these specific bit patterns to recognize the respective direction of the present nystagmus form and to qualify the nystagmus about it (Fig. 2).

### Experimental study design

The experimental study design consisted of two test and two control runs. The first app-test run involved provocation of horizontal nystagmus by irritation of the vestibular organ. This was done by caloric irrigation of both ears with approximately 47° warm water for 80 s. This was followed by recording the nystagmus over 2.5–4 s with the app, for both the right and left ear. First, the detection of the direction of the nystagmus (right, left, up- or down-beat) and second, the detection of the position/ shape of the nystagmus (horizontal, vertical) were recorded with the app. In the first control test, video oculography or video computerized nystagmography was performed during caloric irrigation for detention of the nystagmus. The second app-testing run included provocation of the vertical and horizontal nystagmus by generating an optokinetic sequence nystagmus. This was done with the help of a computer program (playing a series of images), 2 m away from the participants. The standardized procedure automatically determined the frequency of the nystagmus (40°/s and 60°/s) and also the direction, which was detected by the app. The app-test runs have now been subsequently verified by standardized control procedures. In the control test, the eye response and the sequential nystagmus are detected and evaluated by video-computer nystagmography and video-occulography (Table 1).

**Table 1:** Procedure of the test runs

Caloric test	Optokinetic test
Video recording of each test for 2.5–4 s	
Trial 1: rinsing of the right ear canal with warm water (approx. 47 °C) for 80 s	Trail 3: right horizontal nystagmus at 40°/sek
Trial 2: rinsing of the left ear canal with warm water (approx. 47 °C) for 80 s	Trail 4: right horizontal nystagmus at 60°/sek
	Trail 5: left horizontal nystagmus with 40°/sek
	Trail 6: left horizontal nystagmus with 60°/sek
	Trail 7: up-beat vertical nystagmus with 40°/sek
	Trail 8: up-beat vertical nystagmus with 60°/sek
	Trail 0: down-beat vertical nystagmus at 40°/sek
	Trail 10: down-beat vertical nystagmus at 60°/sek

### Statistical analyses

Statistical analyses were performed using GraphPad Prism (version 8, GraphPad Software, La Jolla, CA, USA). Unless otherwise noted, results are given as means with standard deviation (SD) or as absolute values with percentages.

## Results

Twenty patients participated in the study. The patient group consisted of 11 (55%) female and 9 (45%) male participants with a mean age of 25.6 years (± 2.2 SD). Demographic data and test results are shown in Table 2.

**Table 2:** Demographics and tests results for the study group: results presentation of the [direction]—[position] (test results: correct [ +], incorrect [o] or system error [/])

Gender	Age	Eye color	Caloric testing		Optokinetik testing							
			Trial 1	Trial 2	Trial 3	Trial 4	Trial 5	Trial 6	Trial 7	Trial 8	Trial 9	Trial 10
F	23	Green	/	x–x	x–x	x–x	x–x	x–x	o–x	x–x	o–x	x–x
F	27	Brown	/	o–o	x–x	o–x	x–x	x–x	o–x	x–x	o–x	x–x
F	25	Blue	o–x	o–o	x–x	o–x	x–x	x–x	x–x	o–x	x–x	o–o
F	25	Blue	o–o	o–o	x–x	o–x	x–x	x–x	x–x	x–x	x–x	x–x
F	24	Brown	o–o	x–x	x–x	x–x	o–x	x–x	x–x	o–x	x–x	x–x
M	26	Brown	o–x	o–o	x–x	o–x	x–x	x–x	o–x	o–x	x–x	x–x
M	30	Brown	o–x	/	x–x	o–x	x–x	o–x	x–x	x–x	x–x	x–x
F	29	Green	x–x	o–x	x–x	x–x	x–x	o–x	o–x	x–x	o–x	x–x
F	30	Brown	o–x	x–x	x–x	x–x	x–x	x–x	o–x	x–x	o–x	x–x
M	23	Blue	o–o	o–x	x–x	x–x	x–x	x–x	o–x	x–x	x–x	x–x
M	25	Green	o–o	o–x	o–x	x–x	x–x	x–x	o–o	o–o	x–x	x–x
F	23	Blue	o–x	o–x	x–x	o–x	x–x	x–x	x–x	x–x	x–x	o–x
M	26	Blue	o–x	o–x	o–x	o–x	x–x	x–x	o–x	o–x	x–x	x–x
M	24	Brown	o–o	o–x	x–x	o–x	x–x	x–x	x–x	o–o	x–x	o–o
M	22	Brown	o–x	o–x	x–x	x–x	x–x	x–x	x–x	o–x	x–x	x–x
F	27	Blue	o–o	x–x	x–x	x–x	x–x	x–x	x–x	o–x	x–x	x–x
F	26	Green	o–x	x–x	x–x	x–x	x–x	x–x	o–x	o–o	x–x	x–x
F	25	Brown	o–o	/	x–x	x–x	x–x	x–x	o–x	x–x	x–x	x–x
M	26	Brown	o–x	o–x	o–x	o–x	x–x	x–x	x–x	x–x	x–x	x–x
M	26	Blue	o–x	o–x	o–x	x–x	x–x	x–x	x–x	o–x	x–x	x–x

In the first test run (caloric testing), the sensitivity with respect to direction is 15% and position 62.5%. In 10% of the cases, there was a system error. In the second test run (optokinetic testing), the sensitivity with regard to direction is 68.57% and position 95.7%. For test rail 3–10, the sensitivity considered for the individual frequencies is 76.25% for 40°/s and 68.75% for 60°/s (direction) and 98.75% for 40°/s and 93.75% for 60°/s (position). There are no significant correlations between 40 and 60°/s. The results of the two control test procedures show a sensitivity of 100% in all tests. The gold standard diagnostics performed show that the vestibular organ of all subjects is equally excitable on both sides. Both the provoked nystagmus responses to caloric irrigation and the optokinetic consequential nystagmus are too correctly detected and analysed by video-computer nystagmography.

With regard to the question of whether characteristics of the subjects have an influence on the sensitivity of the diagnostics, correlation analyses were performed. The variable age shows a non-significant correlation in the Pearson correlation in relation to result presentation of direction (*r* = − 0.054, *p* = 0.820, *n* = 20) as well as position (*r* = 0.273, *p* = 0.244, *n* = 20). The variable gender shows a significant correlation in relation to result presentation of direction (*r* = − 0.459, *p* = 0.042, *n* = 20) and a non-significant correlation in relation to position (*r* = − 0.076, *p* = 0.749, *n* = 20). The variable eye color shows a non-significant correlation in relation to result presentation of direction (*r* = − 0.012, *p* = 0.969, *n* = 20) as well as position (*r* = 0.127, *p* = 0.594, *n* = 20).

## Discussion

Due to socio-economic change and an increasingly aging population, the healthcare systems of many countries will reach their capacity limits in the future. To enable continuous and rapid diagnostics and meet the growing demand in healthcare, affordable, non-invasive, and easy-to-use healthcare solutions are crucial. However, the benefits of telemedicine are significant in remote areas. However, due to the Covid-19 pandemic, new technology in medicine is also in high demand in urban areas. The increasing prevalence of smartphones combined with modern communication technologies makes them an attractive technology that can provide continuous remote health monitoring and diagnosis in terms of telemedicine at negligible additional cost [2]. Many approaches of medical apps sound promising. However, there is often a lack of sufficient scientific evidence to support a positive impact through use on individual health status. To observe necessary quality criteria in the creation of apps, it is necessary that as many highly qualified health care actors as possible participate in the creation [1].

Several smartphone-based applications are available in the field of otolaryngology [12]. In percentage terms, audiology, sleep medicine, and allergology are the most common. Only 36% of all ENT apps are placed in the field of diagnostics [1]. This shows that there are still opportunities in the field of ENT for the development and use of new media in the context of advancing digitization [2].

The leading symptom "dizziness" is present in clinics and practices worldwide [13]. A quick and sufficient diagnosis supports medical staff in effective decision making to avoid major collateral damage. Previous published studies on balance apps have been concerned with the acquisition and evaluation of the Unterberger treading test, a smartphone-based Rinne test to detect an air-bone-gap or the learning of the liberation maneuvers for positional vertigo [12, 14–16].

In our work, an experimental study design was used to investigate the sensitivity of the developed app analysis for detecting nystagmus in diagnostic decision making in a healthy subject group. Our sensitivity analysis results show that it is possible to diagnose nystagmus and qualify shape and direction using the app. The tests show that the determination of the position works better than that of the direction. One reason for this could be the app's two-stage algorithm system. The first stage of the algorithm distinguishes between horizontal and vertical nystagmus, the second stage of the algorithm analyzes the exact direction. To resize the amount of data, the coding of the first stage is done only in colors. In the second stage this is done with binary codes. The result is a binary bit pattern. The two-stage system is a possible source of error for diagnostics with the app. By reducing the amount of data in the two-stage programming system, the app limits the possibility of correct diagnostics in the case of interfering factors. The limitation could be addressed by optimizing the programming and data processing capabilities.

In addition, the research shows that the sensitivity of the app analysis in terms of direction is significantly lower for caloric testing (15%) than optokinetic testing (68.57%). The main reason for this could be that in the caloric testing, fixation was not removed by Frenzel glasses, as is usually the case. Thus, a fast compensation by the subject is possible. In addition, the time span for filming the nystagmus movement is very short. Here, the usability of the app analysis after caloric testing can thus be discussed. In optokinetic testing, the eyes follow a moving object. It is a natural movement reflex of the eyes which is not pathological, but a normal reaction of the visual system for image stabilization. Optokinetic testing was easier to implement. The speed of the nystagmus did not affect detectability. Our results showed no significant differences in sensitivity with respect to speed on optokinetic testing. However, the visibility of the pattern, the distance of the participants from the screen, and the width of the image pattern to be followed may have an influence on optokinetic nystagmus. This was standardized in our studies. For the caloric test, speeds comparable to the frequency of the optokinetic reflex are given. Furthermore, it could be shown that there is no influence of subject characteristics (gender, age or eye color) on the sensitivity. That the variable "gender" shows a significant correlation with respect to sensitivity (direction) is to be considered coincidental.

If the use of diagnostic apps becomes widespread in practice, it may become problematic if the treating physician and the software come to contradictory results. Care must be taken to ensure that the software is seen only as a supplement to, not a substitute for, the clinical examination [1].

The digital transformation of medicine has been propagated for many years and has already reached medicine for quite some time. The networking of medical care with the aid of modern information and communication technologies is on the rise, most recently driven by the ongoing Covid-19 pandemic. By networking and processing health data, it will be increasingly possible to develop better diagnostic procedures and tailored therapies in the future. New technologies bring great benefits, especially in the areas of prevention, diagnosis, treatment, but also monitoring [4]. Artificial intelligence is the key technology of the future. It helps to master the current challenges of the healthcare system. In addition to increasing the quality of medical care, it will enable an affordable healthcare system. Artificial intelligence promises great success in diagnostics in particular—it ensures that diseases can be detected more precisely and earlier. Driving forward digitization is a key prerequisite for the successful further development of our healthcare system [17].

## Limitation

The present study breaks new ground in analyzing the use of a smartphone app for nystagmus detection. There are some limitations in both the development of the app and the experimental study design that provide suggestions for future research. In the app, the developed algorithm uses a two-stage system for data processing. This two-stage system is a potential source of error for diagnosis using the app. By reducing the amount of data in the two-stage programming system, the app limits its ability to properly diagnose confounding factors. This limitation could be addressed by optimizing the programming and data processing functions. Another limitation of the study is the experimental study design by simulating a pathological nystagmus and the small number of participants. For better validation, appropriate studies need to be performed under clinical conditions in a suitable patient population. This is also necessary for data regarding sufficient decision making.

## Conclusions

The results of our validation study show that it is possible to diagnose nystagmus and determine its shape or direction using the app. Thus, it can be assumed that this experimental smartphone-based nystagmus app for vertigo diagnosis is a useful tool and equally enables fast and effective decision making. The extent to which it can influence and be integrated into decision making in clinical practice now needs to be further investigated.
